# BCG Cell Wall Skeleton As a Vaccine Adjuvant Protects Both Infant and Old-Aged Mice from Influenza Virus Infection

**DOI:** 10.3390/biomedicines9050516

**Published:** 2021-05-05

**Authors:** Ki-Hye Kim, Young-Tae Lee, Yoonsuh Park, Eun-Ju Ko, Yu-Jin Jung, Yu-Jin Kim, Eun-Kyeong Jo, Sang-Moo Kang

**Affiliations:** 1 Center for Inflammation, Immunity & Infection, Institute for Biomedical Sciences, Georgia State University, Atlanta, GA 30302, USA; kihyekim4282@gmail.com (K.-H.K.); leechard75@gmail.com (Y.-T.L.); soopark@coh.org (Y.P.); ej.ko226@gmail.com (E.-J.K.); pharmaco12@gmail.com (Y.-J.J.); yujinsm@gmail.com (Y.-J.K.); 2 Department of Veterinary Medicine, College of Veterinary Medicine and Interdisciplinary Graduate Program in Advanced Convergence Technology and Science, Jeju National University, Jeju 63243, Korea; 3 Department of Immunology and Microbiology, The Scripps Research Institute, La Jolla, CA 92037, USA; 4 Department of Microbiology, College of Medicine, Chungnam NationalUniversity, Munhwa-ro 266, Jungku, Daejeon 35015, Korea; hayoungj@cnu.ac.kr; 5 Infection Control Convergence Research Center, Chungnam National University School of Medicine, Munhwa-ro 266, Jungku, Daejeon 35015, Korea

**Keywords:** influenza virus, split vaccine, cell wall skeleton (CWS) adjuvant, immunological protective immunity

## Abstract

Bacillus Calmette-Guerin (BCG) and the cell wall skeleton (CWS) derived from BCG are known to enhance nonspecific immune activation and anti-cancer immunity; however, their roles as a vaccine adjuvant are largely unknown. Here, we report that BCG-CWS acts as a strong immune adjuvant by promoting the protective immune responses in mouse models with influenza vaccination. The different aged mice immunized with inactivated split vaccine with or without BCG-CWS were challenged with an influenza pandemic virus. When protective immune responses were compared, even a single immunization of adult mice with a BCG-CWS-adjuvanted vaccine showed significantly enhanced humoral immune responses with increased IgG1 and IgG2a isotype antibodies. Importantly, the protective effects by the BCG-CWS adjuvant for influenza vaccination upon humoral and cellular immunogenicity were comparable between infants (6 days and 2 weeks old) and aged (20 months old) mice. Moreover, BCG-CWS dramatically augmented vaccine-mediated protective responses, including decreased viral loads, lung damage, and airway resistance, as well as increased mouse survival, amelioration of weight loss, and proinflammatory cytokine expression in all experimental groups including infant, adults, and old aged mice. We further provided the evidence that the BCG-CWS adjuvant effects were mediated through Toll-like receptors (TLR) 2 and TLR4 signaling pathways. Together, these data suggest that BCG-CWS can be promising as a potential influenza vaccine adjuvant in both young and old aged population through TLR2/4-mediated immune-boosting activities.

## 1. Introduction

An attenuated *Mycobacterium bovis* Bacillus Calmette-Guerin (BCG), a safe and licensed live attenuated vaccine for human tuberculosis, has been implemented in international vaccination programs since the 1920s, with proven safety [1]. BCG vaccination of newborns and infants provides various degrees of protection against human tuberculosis caused by *Mycobacterium tuberculosis* [2]. BCG vaccination and positive tuberculin reaction in early childhood are related to increased survival with nonspecific immune activation and broad protection against other infections [3]. Recent studies suggest that BCG vaccination-induced non-specific immunity and cross-protection are associated with trained immunity, accompanied by epigenetic changes that induce improved antiviral responses in monocytes [4,5]. In a current the coronavirus disease 2019 (COVID-19) pandemic situation, a prior BCG vaccination is also implicated with better clinical outcomes from COVID-19 [6,7].

Thus far, numerous vaccines against emerging and re-emerging infections have limited immunity, which should be improved by an effective immune adjuvant. Different types of adjuvants have been tried in order to increase the prophylactic effects of vaccines against influenza virus infection, which still causes three to five million severe cases of infection globally [8]. Recently, significant efforts have been made to develop new strategies based on universal vaccines and novel adjuvants to overcome the limited efficacy of seasonal vaccine strains against influenza diseases [8,9,10]. For a long time, cell-wall skeletons (CWS) of mycobacteria, which are composed of peptidoglycan attached to arabinogalactan [11], have been considered to possess adjuvant activity [12] with effective antitumor activity [13]. Indeed, BCG-CWS in oil-emulsion has been extensively studied for immunotherapy against various cancers in animal models and in clinical trials [14,15,16,17]. In addition, BCG-derived CWS in an oil-in-water emulsion exhibits the enhanced immunogenicity of recombinant hepatitis B virus protein vaccine [18]. The immune-boosting activity by BCG-CWS appeared to be mediated through its activity on innate immune responses. BCG-CWS is highly effective in dendritic cells (DCs) maturation, secretion of tumor necrosis factor (TNF)-α, interleukin (IL)-6, and IL-12 p40, through stimulation of Toll-like receptor 2 (TLR2) and TLR4 [19]. Blockade of both TLR2 and 4 led to an inhibition of immune activating mechanisms of BCG-CWS in human DC [19,20]. Moreover, a previous study reporting on BCG-CWS with chemical conjugation of model antigens (ovalbumin, KLH, and BSA) sheds light on the usefulness of BCG-CWS as effective adjuvants for universal vaccines against infectious diseases [21].

Aging is usually associated with a susceptibility to infection and development of immune senescence [22,23,24]. Elderly individuals are often high-risk to influenza viral infection and reduced influenza vaccine-specific immune responses, i.e., a difficulty generating protective de novo B and T cells to vaccination [10,23]. The lower efficacy of flu vaccination in the elderly [25,26,27] is attributed to aging-related immune senescence [28] and reduced capacity to generate de novo somatic mutations in IgG upon vaccination [29]. In addition, very young mice with ages up to 7 days (D) and 1–3 weeks (W) old, which are comparable to the stages of developing immune cells in human neonates (≤4 W) and infants (1–12 months old), respectively [30,31], show relatively poorly in generating potent immune responses to vaccination and tends to induce immune tolerance [30,31]. Although it is critical to enhance the vaccine efficacy in the elderly and young individuals, most studies for viral vaccine/adjuvant development have used 6–8 W aged (adolescent) mice, thus it has resulted in a gap between experimental discovery and clinical use. It is essential to develop and optimize the vaccine adjuvant design for both young (neonates/infants) and elderly populations that are more susceptible to infectious agents than healthy adults.

In this study, we investigated BCG-CWS adjuvant effects on influenza vaccine efficacy in neonatal infant ages (6 D, 2 W), adult, and old (20 M) age mice after priming and/or boosting by intramuscular vaccination with an inactivated split vaccine prior to challenge with influenza virus infection. Surprisingly, we found that BCG-CWS-adjuvanted influenza vaccination significantly enhanced humoral and cellular responses as well as protective efficacy in lethal dose influenza viral infection models with both neonatal/infant and old-aged mice. The immune activation by BCG-CWS was, at least, partly mediated in DCs through TLR2 and TLR4. Taken together, our results show that BCG-CWS adjuvant can efficiently augment protective immune activities by influenza vaccine, even against severe influenza infection, regardless of age. BCG-CWS-induced enhancement of innate immune protection may provide a promising strategy for developing effective vaccination in susceptible populations, in whom the conventional vaccines are only modestly effective or ineffective depending on the ages.

## 2. Materials and Methods

### 2.1. Animals, Vaccine, and Reagents

Female BALB/c mice (six-weeks old, 6 W) and retired breeders of BALB/c mice (7–9 months old) were purchased from the Taconic Farms (Hudson, NY, USA). Retired BALB/c mice were aged to become 20 months old (20 M) in the animal facility at the Georgia State University (GSU). Neonate (6 days old, 6 D) and infant (2 weeks old, 2 W) BALB/c mice were obtained in the mouse breeding animal facility at GSU. Animal experiments were performed under the guidelines of the approved IACUC protocol (A21004). We propagated A/California/04/2009 (H1N1, A/Cal) influenza virus using embryonated chicken egg substrates. Inactivated influenza split vaccine (sCal, S) derived from the 2009 pandemic strain of A/California/07/2009 (H1N1) virus kindly provided by Green Cross (Yongin-si, Gyeonggi-do, Korea).

### 2.2. Preparation of BCG-CWS

CWS from *Mycobacterium bovis* BCG (Pasteur 1173P2) was prepared as previously described [21], with slight modification. Briefly, the wet cell mass of *M. bovis* BCG inactivated by autoclave was suspended with five volumes of 2% (*w/v*) Triton X-100, then sonicated for 5 min using a VCX 750 ultrasonic processor (Sonics & Materials, Inc., Newtown, CT). The homogenate sample was heated in a boiling water bath for 3 h, then pelleted by centrifugation at 15,344× *g* for 30 min. The sample was repeated twice with 2% (*v/v*) Triton X-100 and three-times with 2% (*w/v*) sodium dodecyl sulfate (SDS) for delipidation and deproteination. The BCG-CWS pellet was washed twice with five volumes of 10% (*v/v*) isopropyl alcohol and three times with five volumes of acetone to remove the SDS by vigorous mixing and centrifugation. Finally, purified BCG-CWS was resuspended in 100% isopropyl alcohol and stored in a -20 °C freezer until use. The dry weight of the CWS was calculated by pelleting at 14,000 rpm for 10 min and then drying overnight at 50 °C.

### 2.3. Immunization and Infection

BALB/c mice (6 W, n = 20 per group) were intramuscularly (i.m.) prime immunized with sCal (S, 0.3 µg) with or without BCG-CWS (25 µg). The groups of 6 D, 2 W, and 20 M age BALB/c mice (*n* = 18 or 10 per group) were intraperitoneal (i.p.) prime for 6 D age infant mice or i.m. prime (2 W or older) and boost immunized with sCal (S) with or without BCG-CWS. Blood samples were collected at 2 weeks after prime and boost immunization to determine antibody responses.

To investigate the protective efficacy and immunological profiles after challenge, immunized mice were challenged with A/Cal (H1N1) virus at a lethal dose of 2 × LD_50_ (50% mouse lethal dose) equivalent to 3 × 10^3^ EID_50_ (50% embryo infectious dose) after prime or prime-boost immunization. After challenge, body weight changes and survival rates were monitored for 14 days. Another set of mice group was monitored to determine enhanced pause (PenH) of air inhalation by using whole body plethysmography (emka Technologies, Sterling, VA, USA) and euthanized day 7 post infection (7 dpi) to collect bronchoalveolar lavage (BAL) fluids (BALF) and lung and spleen tissues.

### 2.4. ELISA and ELISPOT Assay

Virus antigen specific antibody responses were determined in immune sera collected after immunization in BALF and lung lysates harvested at day 7 post infection (dpi) by enzyme-linked immunosorbent assay (ELISA) as previously described [32]. Briefly, serially diluted immune sera were applied to a 96-well plate precoated with inactivated A/Cal virus (iA/Cal, 20 µg/mL per well). IgG and IgG isotype antibodies were quantified by horseradish peroxidase (HRP)-conjugated anti-mouse IgG, IgG1, IgG2a (Southern Biotech, Birmingham, AL, USA), and tetramethylbenzidine (eBioscience, San Diego, CA, USA) as a substrate. The cytokine levels including interleukin 6 (IL-6), tumor necrosis factor alpha (TNF-α), and interferon gamma (IFN-γ) were measured in lung lysates harvested at 7 days post infection (dpi) using ELISA kits (eBioscience, San Diego, CA, USA) according to the manufacturer’s instructions.

To determine IFN-γ cytokine secreting cells, lung (2 × 10^5^) and splenocytes (10^6^) were cultured on 96-well enzyme-linked immunospot (ELIspot) plates precoated with anti-mouse IFN-γ monoclonal capture antibody (BD Biosciences, San Diego, CA, USA) in the presence of inactivated A/Cal virus (iA/Cal, 4 µg/mL). The cytokine spots were developed with biotinylated anti-mouse IFN-γ detection antibody and alkaline phosphatase-labeled streptavidin (BD Pharmingen, San Diego, CA, USA). The spots were visualized with a 3,3′-diaminobenzidine substrate and counted by an ELSPOT reader (BioSys, Miami, FL, USA).

### 2.5. Hemagglutination Inhibition (HAI) Assay

HAI assay was performed as previously described [33]. Immune sera were treated with receptor destroying enzymes (RDE, Sigma Aldrich, St. Louis, MO, USA) and then incubated at 37 °C for 16 h. The samples were heat inactivated at 56 °C for 30 min. Serially 2-fold diluted sera were incubated with 4 HA units of A/Cal for 30 min, then admixed with 0.5% chicken red blood cells (RBC, Lampire Biological Laboratories, Pipersville, PA, USA) to determine HAI titers.

### 2.6. Virus Titration

The lung samples were harvested at day 7 after A/Cal infection. Lung viral titers were determined in embryonated chicken eggs (Hy-Lin North America, LLC., Mansfield, GA, USA). Briefly, lung lysates were serially diluted 10-fold and injected into 10-day embryonated chicken eggs and then incubated at 37 °C for 3 days. The titers were determined by hemagglutination assay of the allantoic fluids. The 50% embryo infectious dose (EID_50_) was evaluated according to the Reed and Muench method [34].

### 2.7. Flow Cytometry

For cellular phenotypic analysis in BAL and lung cells, the single cells were prepared and stained with fluorophore-labeled antibodies specific for anti-mouse CD45 (clone 30-F11, BD Pharmingen, San Diego, CA, USA), CD11b (clone M1/70, BD), CD11c (clone N418, eBioscience, San Diego, CA, USA ), F4/80 (clone BM8, eBioscience, San Diego, CA, USA), Ly6c (clone HK1.4, BioLegend), Siglec F (clone E50-2440, BD), MHC class II (clone M5/114.15.2, BioLegend, San Diego, CA, USA), CD103 (clone 2E7, BioLegend, San Diego, CA, USA), and CD16/32 (clone 14-0161-85, eBioscience, San Diego, CA, USA). Live lymphocyte populations were first gated by forward versus side scatter strategic gating to exclude small dead cells and cell debris, followed by the next gating of CD11b^+^ and CD11b^-^ granulocytes and CD3^+^ T lymphocytes.

For antigen-specific T cell responses, the cells from the airway, lung, and spleen were in vitro stimulated with iA/Cal virus (4 µg/mL) at 37 °C. After 5 h stimulation, the lymphocytes were stained with anti-mouse CD4 (clone 553051, BD, San Diego, CA, USA) and CD8 (clone 25-0081-82, eBioscience, San Diego, CA, USA) monoclonal antibodies. BD Cytofix/Cytoperm^TM^ Plus kit was used to fix and permeabilize specific marker labelled lymphocytes, and cytokine positive cells were stained with anti-mouse IFN-γ (clone XMG1.2, BD). All samples were analyzed on a Becton-Dickinson LSR-II/Fortessa flow cytometer (BD biosciences, San Jose, CA, USA) and analyzed by using Flowjo software (Tree Star Inc., Ashland, OR, USA).

### 2.8. Preparation of BMDMs and BMDCs

Primary bone marrow derived macrophages (BMDMs) from wild type BALB/c and C57BL6 (Jackson laboratory, Bar Harbor, ME, USA) were generated as described previously [35]. For differentiation of macrophages, the bone marrow cells were cultured in Dulbecco’s modified Eagle’s medium (DMEM, Corning, Tewksbury MA, USA) containing 10% fetal bovine serum (FBS), penicillin (100 IU/mL), and streptomycin (100 µg/mL) in the presence of mouse macrophage colony-stimulating factor (mM-CSF, R&D Systems) for 5~6 day. Then, the BMDMs were stimulated with 10 µg/mL of BCG-CWS for 24 h.

For BM-derived dendritic cells (BMDCs) preparation, primary bone marrow cells were harvested from wild type (WT, BALB/c and C57BL6), TLR-2 knockout (KO, B6.129*Tlr*^2tm1Kir^/J, Jackson laboratory), or TLR-4 KO (B6.B10ScN-*Tlr4^lps-del^*/JthJ, Jackson laboratory, Bar Harbor, ME, USA). Preparation of BMDCs was done as described previously [36]. Briefly, BM cells from WT and KO mice were cultured in 2ml of complete media of Roswell Park Memorial Institute (RPMI) 1640 (Corning, Tewksbury MA, USA) including 10% FBS and penicillin (100 IU/mL) and streptomycine (100 µg/mL) supplemented with mouse granulocyte-macrophage colony-stimulating factor (mGM-CSF, R&D Systems, Minneapolis, MN, USA) in 6-well plates. Starting at the third day of culture, half of the medium was replaced with fresh prewarmed medium in presence of mGM-CSF. After 6 to 8 days culture, loosely adherent cells were harvested and washed with complete medium. The cells were seeded into 96-well plate with 2 × 10^5^ cells per well and were stimulated with BCG-CWS (10 µg/mL) or medium control for 24 h. The culture supernatants were used to determine the levels of cytokines.

### 2.9. Statistical Analysis

All results are presented as the mean ± the standard errors of the mean (SEM). The statistical significance for all experiments was performed by one- or two-way analysis of variance (ANOVA). Prism software (GraphPad Software, Inc., San Diego, CA, USA) was used for all data analysis. The comparison used to generate a *p* value is indicated by horizontal lines (*; *p* < 0.05, **; *p* < 0.01, ***; *p* < 0.001).

## 3. Results

### 3.1. BCG-CWS Enhances the Adjuvant Effects on Heightening Humoral Responses and Protective Efficacy in Adult Mice After Single Dose Influenza Vaccination

**Figure 1:**
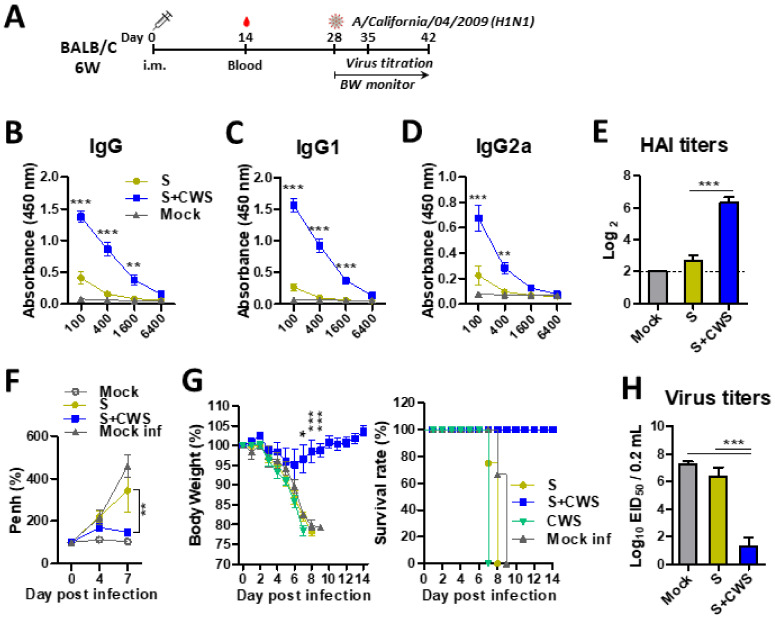
BCG-CWS exhibits significant adjuvant effects on enhancing IgG, HAI, and protective efficacy in adult mice after single dose influenza vaccination. (**A**) Immunization schedule (*n* = 20). 6-week-old (6 W) BALB/c mice were intramuscular (i.m.) immunized either with sCal (S) or S+CWS. CWS: BCG-CWS. Blood samples were collected at 2 weeks after prime. (**B**–**D**) Antibody responses specific for inactivated A/Californial/04/2009 virus (iA/Cal) in prime immune sera. € Hemagglutinin inhibition titers (HAI) to A/California/04/2009 (A/Cal) were determined in prime immune sera. (**F**–**H**) Immunized mice were infected with A/Cal virus at a dose of 2 × LD_50_ (50% mouse lethal dose) equivalent to 3 × 10^3^ EID_50_ (50% embryo infectious dose) at 4 weeks after prime. PenH (**F**) and body weight changes or survival rates (**G**) were monitored for 7 days and 14 days (*n* = 10), respectively. (**H**) Lung viral titers were determined by an egg inoculation assay at 7 days post infection (*n* = 10). Mock: PBS control and no infected, S: split sCal vaccine only, S+CWS: split plus CWS, CWS: BCG-CWS adjuvant only, Mock inf: PBS and infected with A/Cal virus. Statistical significance was calculated by using one- or two-way ANOVA and a Bonferroni’s multiple-comparison test. Error bars indicate the mean ± SEM. *; *p* < 0.05, **; *p* < 0.01, ***; *p* < 0.001.

Our previous study reported that adult mice immunized with inactivated virus vaccine without adjuvants in twice (prime and boost) induced protection against homologous A/Cal/2009 virus [37]. Thus, it was expected that CWS would have prominent adjuvant effects in a single dose vaccination with inactivated influenza virus in adult BALB/c mice. To determine whether BCG-CWS exhibits the adjuvant effects on the efficacy of influenza vaccination, the groups of 6 W-aged BALB/c mice were i.m. primed with inactivated split influenza virus (A/California/04/2009, H1N1) vaccine (sCal, 0.3 µg) in the presence or absence of BCG-CWS (Figure 1A). At 2 weeks after prime dose, the BCG-CWS-adjuvanted sCal vaccine group induced significantly higher levels of virus specific IgG, IgG1, and IgG2a isotype and HAI antibodies than those in the vaccine alone group (Figure 1B–E). When the naïve and vaccinated mice were challenged with a lethal dose of homologous A/Cal (H1N1) virus at 4 weeks after prime dose, the split vaccine alone and unvaccinated naïve groups, but not the BCG-CWS plus vaccine group, displayed significantly high resistance in air inhalation as presented by PenH values (Figure 1F). Consistently, BCG-CWS-adjuvanted single vaccination induced protection with a minimum weight loss (~5%), whereas the unvaccinated naïve (mock), CWS adjuvant only without vaccine (CWS), and split sCal only vaccine control groups were not protected (Figure 1G). Lung viral titers were close to the detection limit by lowering 4 log10 magnitudes in the BCG-CWS-adjuvanted vaccine group (Figure 1H). Significantly higher lung viral titers by 100,000-fold were detected in the naïve or sCal vaccine only groups compared to BCG-CWS adjuvanted vaccination (Figure 1H). These results support the significant adjuvant effects of BCG-CWS on enhancing the immunogenicity and efficacy of influenza vaccination.

### 3.2. BCG-CWS-Adjuvanted Boost Vaccination Enhances IgG and HAI Titers Against Viral Antigen in Both 6 D-Aged (Neonatal) and 2 W-Aged (Infant) Mice

**Figure 2:**
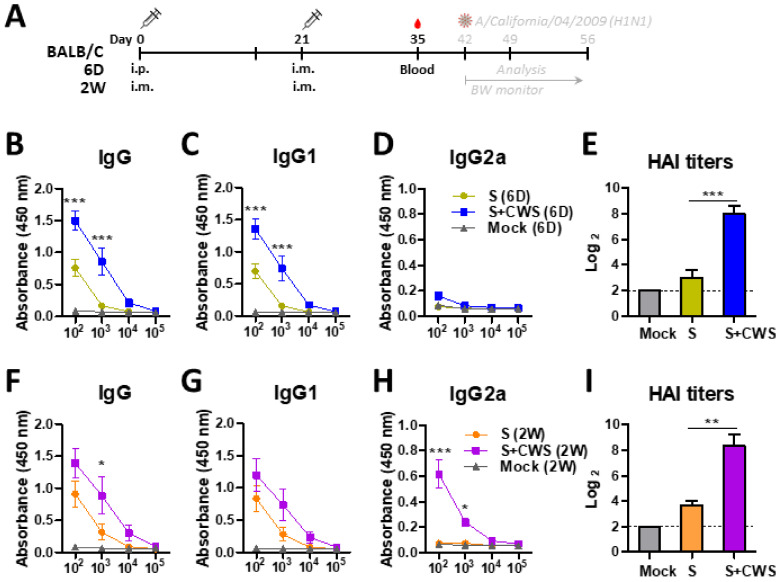
IgG antibody responses and HAI titers in BCG-CWS adjuvanted vaccination in neonatal (6 D) or infant (2W) age mice. (**A**) Immunization schedule. BALB/c mouse pups (*n* = 18) at 6 days (6 D) and 2 weeks (2 W) age were i.p./i.m. or i.m./i.m. immunized either with sCal (S) vaccine or S+CWS (CWS: BCG-CWS) at a 3-week interval. (**B**–**D**) Serum IgG, IgG1, and IgG2a specific for virus antigen in 6 D age mice, and (**F**–**H**) 2 W age mice. (**E**,**I**) HAI titers against A/Cal in boost sera of 6 D and 2 W age mice. Statistical significance was calculated by using one- or two-way ANOVA and a Bonferroni’s multiple-comparison test. Error bars indicate the mean ± SEM. *; *p* < 0.05, **; *p* < 0.01, ***; *p* < 0.001.

It is significant to enhance the efficacy of vaccination in neonates and infants when most vaccinations are administered. Neonatal (6 D) and infant (2 W) mice were prime boosted with sCal vaccine (0.3 µg) +/− BCG-CWS in a 3-week interval, and then serum IgG levels were determined after boost (Figure 2A). BCG-CWS administration significantly enhanced the levels of IgG and IgG1, but not IgG2a isotypes, in the neonatal group after vaccination (Figure 2B–D). The impact of BCG-CWS adjuvant on enhancing HAI titers was also significant in the S+BCG-CWS group by an increase of over 30-fold, compared to the sCal (S) only group (Figure 2E). In addition, the infant group with BCG-CWS-adjuvanted vaccination induced approximately 10-fold higher levels of IgG, IgG1, and IgG2a antibodies, as well as 24-fold higher HAI titers, than those in vaccine alone 2 W mice (Figure 2F–I). These results support significant adjuvant effects of BCG-CWS on enhancing IgG and HAI titers in neonatal and infant mice with vaccination.

### 3.3. BCG-CWS Significantly Increases Protective Efficacy in Vaccinated Neonatal and Infant Mice

**Figure 3:**
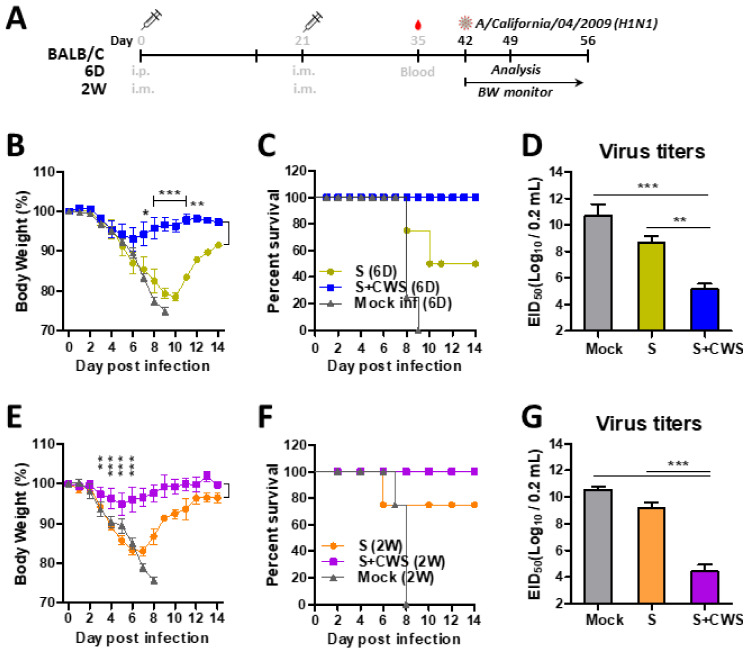
BCG-CWS adjuvant effects on enhancing protective efficacy in vaccinated 6 D and 2 W age mice. (**A**) Challenge plan of immunized mice. Vaccinated 6 D and 2 W groups were challenged with A/Cal virus (2 × LD_50_) at 3 weeks after boost. (**B**,**C**) Body weight changes and survival rates in 6 D age mice (*n* = 8). (**E**,**F**) Body weight changes and survival rates in 2 W age mice (*n* = 8). (**D**,**G**) Lung viral titers were determined by an egg inoculation assay at 7 dpi (*n* = 10). Statistical significance was calculated by using one- or two-way ANOVA and a Bonferroni’s multiple-comparison test. Error bars indicate the mean ± SEM. *; *p* < 0.05, **; *p* < 0.01, ***; *p* < 0.001.

At 3 weeks after boost, the neonatal and infant mouse groups were challenged with a lethal dose of A/Cal virus. As expected, naïve mice (Mock) markedly increased the mortality upon challenge with virus infection (Figure 3). However, the 6 D-aged mice with S+BCG-CWS vaccination exhibited significantly less weight loss (~8%) and were totally protected from lethality (100% survival rates), whereas the vaccinated only group (S vaccine) lost weight over 20% and survival rates were ~50% (Figure 3B,C). In addition, the S+BCG-CWS 6 D group showed lower viral titers by over 2000-fold than those in the S only 6 D group (Figure 3D). Consistently, the S+BCG-CWS formulation in 2 W-aged mice displayed minimum weight loss (~5%), compared to those with the S only that showed ~18% weight loss, suggesting a partial protection (Figure 3E,F). Furthermore, the lung viral titers in 2 W-aged mice between the S+BCG-CWS and the S groups were significantly different by 5 log_10_ magnitudes (Figure 3G), indicating a dramatic protective effect by BCG-CWS adjuvanted vaccination.

**Figure 4:**
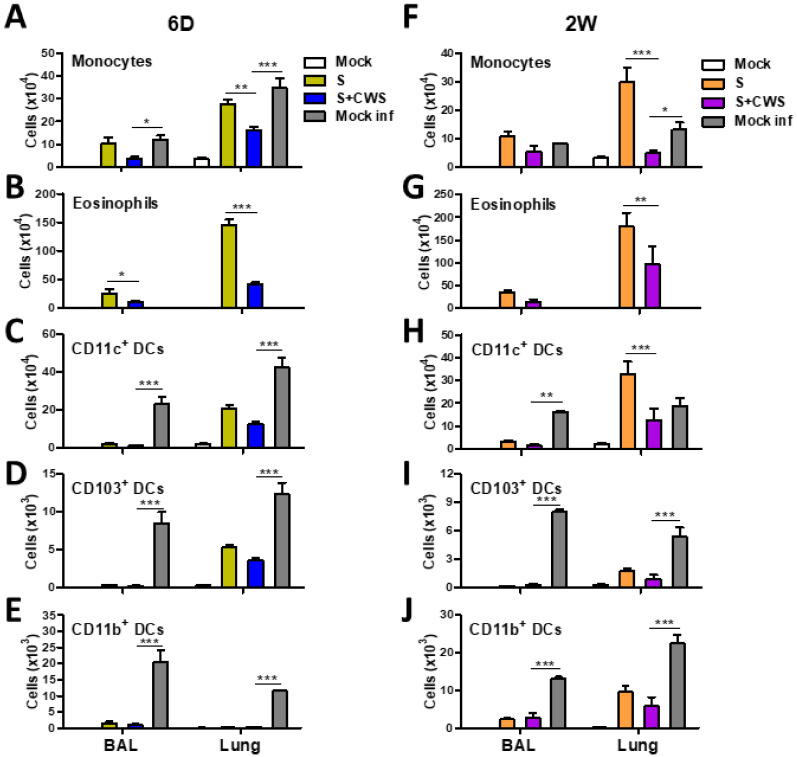
BCG-CWS-adjuvanted 6 D and 2 W mice protect against excessive cellular infiltration into the lung after challenge**.** Cellular phenotypes were determined by flow cytometry in BAL and lung samples collected at 7 dpi with A/Cal virus. (**A**,**F**) Monocytes (^+^CD11b^+^Ly6c^hi^F4/80^+^) and (**B**,**G**) Eosinophils (^+^CD11b^+^CD11c^-^SiglecF^+^) were gated from CD11b^+^ cells. CD11c^+^ dendritic cells (DCs, CD45^+^F4/80^-^CD11c^+^ MHCII^hi^), CD103^+^ DCs (CD45^+^F4/80^-^CD11c^+^MHCII^hi^CD11b^-^CD103^+^), and CD11b^+^ DCs (CD45^+^F4/80^-^CD11c^+^MHCII^hi^CD11b^+^CD103^-^) were gated from CD45^+^F4/8^-^ cells (**C**,**H**) and CD45^+^F4/80^-^CD11c^+^MHCII^hi^ cells (**D**–**J**), respectively, in BAL and Lung of 6 D and 2 W age mice (*n* = 10 each age mice). The low cell numbers of eosinophils were observed to be 1030 and 3292 in the BAL and lungs from mock infected mice. Statistical significance was calculated by using two-way ANOVA and a Bonferroni’s multiple-comparison test. Error bars indicate the mean ± SEM. *; *p* < 0.05, **; *p* < 0.01, ***; *p* < 0.001.

On day 7 post influenza virus infection, the naïve 6 D and 2 W groups showed high levels of cellular infiltrates as determined by flow cytometry, including monocytes and different DC subsets (CD11c^+^ DCs, CD103^+^ DCs, and CD11b^+^ DCs), in the lungs and airway BALF (Figure 4). The sCal split vaccine alone showed high levels of monocytes and eosinophils in the lungs and BALF from 6 D-aged mice (Figure 4A,B), and high levels of monocytes, eosinophils, and CD11c^+^ DCs in the lungs from 2 W-aged mice, after challenge with influenza virus (Figure 4F–H). Notably, BCG-CWS adjuvanted sCal split vaccination significantly ameliorated infiltration of monocytes in the lungs, as well as eosinophils in both BALF and lungs from 6 D-aged mice, when compared with sCal split vaccination only (S) did (Figure 4A,B). In addition, BCG-CWS+S vaccination markedly abrogated the infiltration of monocytes, eosinophils, and CD11c^+^ DCs, in the lungs from 2 W-aged mice, when compared with sCal split vaccination only (S) did (Figure 4F–H). Moreover, BCG-CWS adjuvanted sCal split vaccination showed a significant reduction in infiltrating inflammatory cells such as monocytes, CD11c^+^ DCs, CD103^+^ DCs, and CD11b^+^ DCs in both BALF and lungs from 6 D-aged mice, when compared with those from naïve infected mice (Figure 4C–E). In 2 W-aged mice, BCG-CWS adjuvanted sCal split vaccination significantly inhibited the monocytes, CD103^+^ DCs, and CD11b^+^ DCs in the lungs as well as CD11c^+^ DCs, CD103^+^ DCs, and CD11b^+^ DCs in BALF, when compared with those from naïve infected mice (Figure 4F–J). A similar pattern was observed in the frequency for inflammatory cells and antigen presenting cells infiltrated (Supplementary Figure S1). These data strongly suggest that BCG-CWS adjuvanted vaccination prevents inflammatory cellular infiltrates in the airways and lungs after lethal infection, thus providing efficient adjuvant effects by enhancing the protective efficacy against lethal influenza virus infection even in 6 D- and 2 W-aged mice.

### 3.4. BCG-CWS Adjuvant Plays a Critical Role in Enhancement of the Immunogenicity and Efficacy of Influenza Vaccination in 20 M-Aged Mice

**Figure 5:**
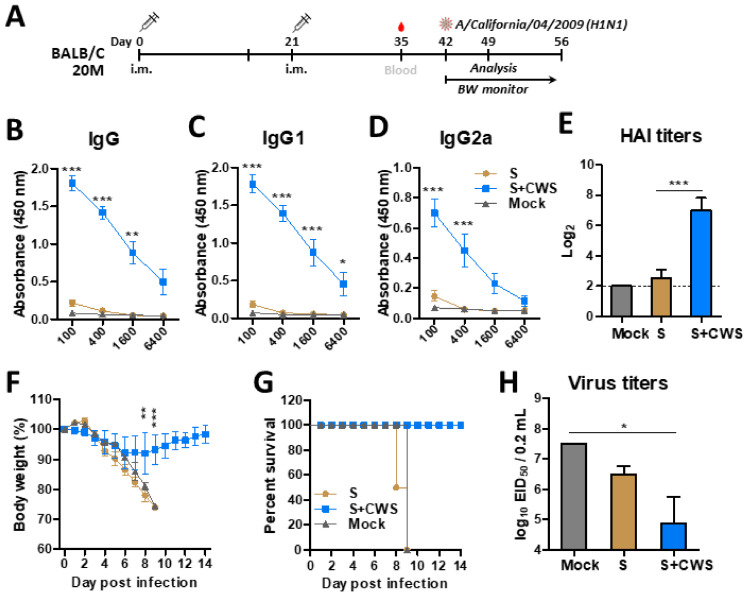
BCG-CWS adjuvant effects on enhancing IgG, HAI, and protective efficacy in vaccinated aged (20 M) mice. (**A**) Immunization and challenge schedule. The groups of aged (20 M) mice (*n* = 10) were i.m. twice immunized with sCal (S) or S+CWS (CWS: BCG-CWS) at a 3-week interval. (**B**–**D**) Serum IgG, IgG1, IgG2a specific for virus antigen in boost sera. (**E**) HAI titers against A/Cal virus in boost sera. (**F**,**G**) Weight changes and survival rates after lethal dose A/Cal virus infection in aged (20 M) mice (*n* = 5). (**H**) Lung viral titers (*n* = 5) at 7 dpi. Statistical significance was calculated by using one- or two-way ANOVA and a Bonferroni’s multiple-comparison test. Error bars indicate the mean ± SEM. *; *p* < 0.05, **; *p* < 0.01, ***; *p* < 0.001.

To determine the potential effects of BCG-CWS adjuvants upon enhancement of virus-specific IgG antibodies in elderly groups, the 20 M-aged mice were prime-boost vaccinated with sCal (0.3 µg) with or without BCG-CWS at a 3-week interval (Figure 5A). It was noted that BCG-CWS adjuvanted split vaccination dramatically enhanced virus-specific IgG, IgG1, and IgG2a isotypes as well as HAI titers (~20-fold) in 20 M-aged mice (Figure 5B–E). The split vaccine alone did not induce significant levels of virus-specific IgG antibodies and HAI titers in 20 M-aged mice, suggesting that BCG-CWS adjuvants are even more critical in increasing IgG antibody responses to vaccination in these aged group.

When both unvaccinated and vaccinated 20 M mice were challenged with a lethal dose A/Cal (H1N1) virus, BCG-CWS adjuvant-vaccinated mice were more protected against lethal infection, followed by low to moderate (~8%) weight loss, than the vaccination only or mock control groups (Figure 5F,G). It was approximately 100-fold lower lung viral titers at day 7 post challenge in the S+BCG-CWS group in 20 M-aged mice, than those in the vaccine only or mock control groups (Figure 5H). In contrast, the vaccination alone did not protect the 20 M-aged mice against A/Cal challenge, exhibiting severe weight loss and 0% survival rates, similar to those observed in the mock control (Figure 5F,G). Together, BCG-CWS acts as a critical adjuvant through enhancement of the vaccine protective efficacy in aged mice.

### 3.5. BCG-CWS-Adjuvanted Influenza Vaccination Attenuates Inflammatory Cytokine Production While Promotes Virus-Specific IgG Responses in the Lung After Infection

**Figure 6:**
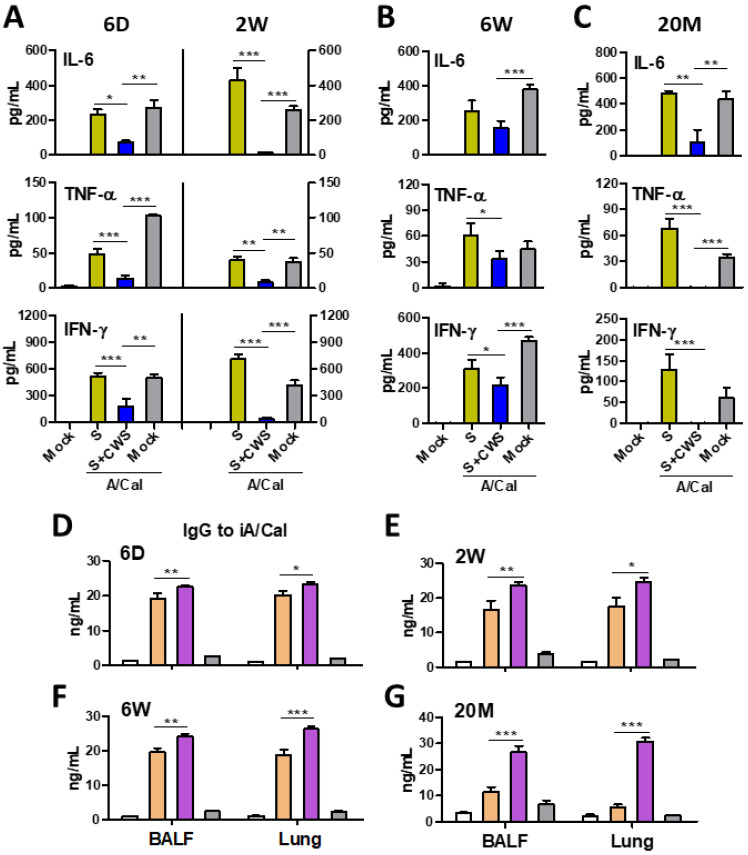
Inflammatory cytokines and virus specific IgG antibody responses in lung samples from vaccinated mice after infection. BALF and lung lysates were collected at 7dpi after challenging of immunized mice (*n* = 10 for 6 D, 2 W, and 6 W mice, *n* = 5 for 20 M mice) to evaluate protective efficacy. The levels of cytokine and antibody were determined by ELISA. (**A**) Inflammatory cytokines (IL-6, TNF-α, and IFN-γ) in lung samples collected from 6 D or 2 W mice with prime-boost. (**B**) Lung inflammatory cytokines from 6 W adult mice with prime. (**C**) Lung inflammatory cytokines from 20 M aged mice with prime-boost. (**D**–**G**) Virus specific IgG antibody levels in BALF and lung samples collected at 7 dpi from the 6 D, 2 W, 6 W, and 20 M mouse groups. Statistical significance was calculated by using one-way ANOVA and a Dunnett’s multiple-comparison test. Error bars indicate the mean ± SEM. *; *p* < 0.05, **; *p* < 0.01, ***; *p* < 0.001.

It is an important parameter to protect against inflammation due to viral infection. After virus challenge, high levels of inflammatory cytokines (IL-6, TNF-α, and IFN-γ) were detected in the lungs from the mock and split vaccine alone groups immunized at infant (6 D, 2 W), adult (6 W), and aged (20 M) ages (Figure 6A–C). Inflammatory cytokines appeared to be correlating with severity of weight loss and high lung viral titers (data not shown). The BCG-CWS-adjuvanted (S+BCG-CWS) vaccination effectively attenuated the production of inflammatory cytokines including IL-6, TNF-α, and IFN-γ, in the lungs from all aged mouse groups (6 D, 2 W, 6 W, and 20 M) after lethal infection (Figure 6A–C).

Mucosal antibodies play a role in the protection during respiratory viral infection [38,39]. We next determined virus-specific IgG levels in BALF and lung extracts at 7 days after infection. The mock treatment did not induce virus-specific IgG antibodies at detectable levels in BALF and lungs from naïve control mice (Figure 6D–G). However, the split vaccine +/− BCG-CWS groups showed high levels of virus specific IgG antibodies in the BALF and lung from all experimental groups (6 D, 2 W, and 6 W old; except 20 M), after infection (Figure 6D–G). It was noted that IgG mucosal responses were significantly increased by BCG-CWS adjuvanted vaccination in lungs and BALF from all experimental groups (6 D, 2 W, 6 W, and 20 M), compared with those by vaccination alone (Figure 6D–F). In 20 M-old mice, the BCG-CWS adjuvanted vaccination, but not split vaccine alone, induced virus specific IgG antibodies at significant levels in the BALF and lung extracts at day 7 post challenge (Figure 6G). Collectively, these data strongly suggest that BCG-CWS-combined influenza vaccination attenuates pathological inflammatory responses, but promotes protective IgG mucosal immune responses, during viral infection.

### 3.6. BCG-CWS-Adjuvanted Vaccination Induces IFN-γ-Secreting T Cell Responses at Systemic and Mucosal Sites from Young to Old Aged Mice

**Figure 7:**
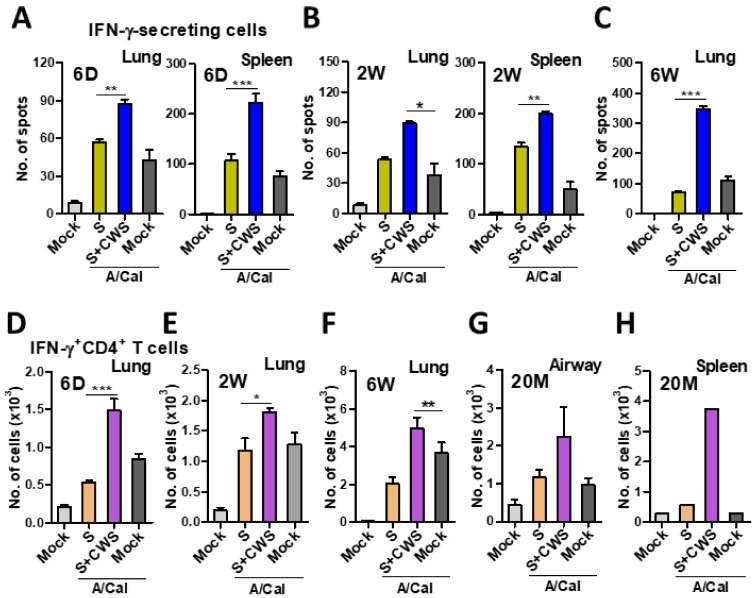
IFN-γ secreting cellular responses in lung and spleens at early time after challenge. IFN-γ secreting cells were determined in lung and spleen samples collected from the 6 D, 2 W, 6 W, and 20 M groups at 7 dpi with A/Cal virus challenge. (**A**–**C**) Virus antigen-specific IFN-γ secreting cells in the lung or splenocytes from the different age vaccinated groups as determined by ELISpot assay. (**D**–**H**) The IFN-γ-secreting CD4^+^ T cells (IFN-γ^+^CD4^+^ T cells) in the lung, airway (BAL), or spleens from the different age vaccinated groups as determined by intracellular cytokine staining and analyzed by flow cytometry. Statistical significance was calculated by using one-way ANOVA and a Dunnett’s multiple-comparison test. Data are presented from all animals. Error bars indicate the mean ± SEM. *; *p* < 0.05, **; *p* < 0.01, ***; *p* < 0.001.

We next determined the protective IFN-γ secreting cell responses at systemic (spleen) and mucosal (lung) sites from young infant to old age mice at 7 days after challenge (Figure 7). IFN-γ-secreting cells were significantly increased in the spleen and lungs from the 6 D- and 2 W-aged mice with BCG-CWS adjuvanted vaccination, when compared to those mice with vaccination only or mock control (Figure 7A,B). As for 6 W adult mice, IFN-γ secreting lung cell spots were detected at over 3-fold higher levels in the BCG-CWS adjuvanted vaccine group, compared to the mock control or vaccine only group (Figure 7C).

We further determined the BCG-CWS adjuvant effects on inducing IFN-γ-secreting CD4^+^ T cell responses by intracellular cytokine staining analyzed by flow cytometry assays (Figure 7D–H). The levels of IFN-γ^+^ CD4^+^ T cells were significantly higher in the lungs from the 6 D- and 2 W-aged mice with BCG-CWS adjuvanted vaccination than those in the vaccine only group (Figure 7D,E). In addition, 6 W-aged mice with BCG-CWS adjuvanted vaccine induced IFN-γ^+^ CD4^+^ T cells in the lungs at higher levels than those with the vaccine alone group (Figure 7F). Mock control of 6 D-, 2 W-, and 6 W-aged mice showed substantial increase of lung IFN-γ^+^ CD4^+^ T cells as a result of live virus infection (Figure 7D–F). In 20 M-aged mice, the BCG-CWS adjuvanted vaccine markedly increased IFN-γ^+^ CD4^+^ T cells at high levels in their airway BALF and spleen cells, compared with those from mock control mice (Figure 7G,H). These data collectively suggest that IFN-γ-producing T cells were significantly increased in all aged groups of mice by BCG-CWS adjuvanted vaccination.

### 3.7. BCG-CWS Stimulate Proinflammatory Cytokine Production in Antigen-Presenting Cells Through TLR2 and TLR4 Pathways

**Figure 8:**
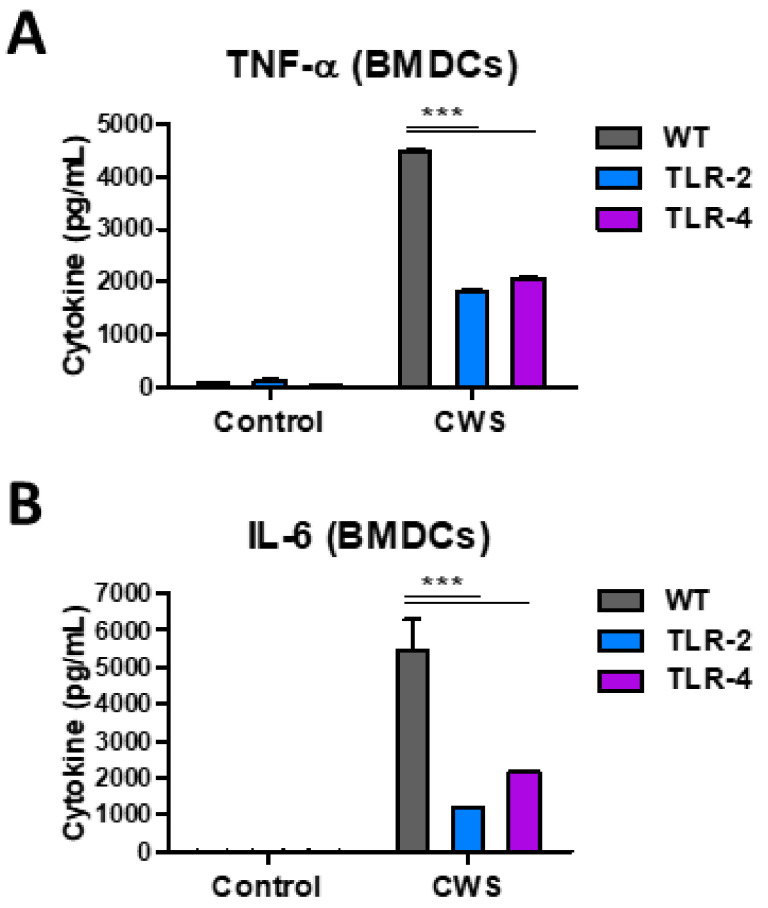
BCG-CWS stimulate antigen presenting cells to secrete cytokines via TLR-2 and TLR-4 pathways**.** Primary BMDCs isolated from WT (C57BL/6), TLR-2 KO, or TLR-4 KO mice were stimulated with BCG-CWS (10 µg/mL) or medium control for 24 h. (**A**,**B**) The data represent the concentrations of TNF-α and IL-6 in the BMDCs culture supernatants. Error bars indicate the mean ± SEM. ***; *p* < 0.001.

To better understand the adjuvant actions of BCG-CWS, antigen presenting cells (APCs), such as BMDCs and BMDMs, were subjected to examination of cytokine production after in vitro cultures (Supplementary Table S1). Inflammatory cytokines (TNF-α and IL-6) were secreted at high levels by both BMDMs and BMDCs after stimulation with BCG-CWS (10 µg/mL). Th1-promoting IL-12 and anti-inflammatory IL-10 cytokines were produced at higher levels by BMDCs than those by BMDMs after BCG-CWS stimulation (Supplementary Table S1). In addition, we cultured BMDCs from TLR2 and TLR4 knockout mice and measured cytokine levels after BCG-CWS stimulation. Notably, BMDCs from TLR2 and TLR4 knockout mice significantly reduced the production of TNF-α and IL-6 than those from wild type mice did, after BCG-CWS stimulation (Figure 8A,B). These results suggest that BCG-CWS exhibits stimulating effects in APCs through induction of inflammatory cytokines via TLR2 and TLR4 signaling pathways.

## 4. Discussion

Vaccine effects, preponderance of infection, and pathological inflammatory phenotypes depend on age. The enhancement of vaccine effectiveness in neonates, infants, and elderly populations should be of high priority, considering the increased susceptibility to pathogenic infections in these populations. To our knowledge, this is the first report to show that BCG-CWS is a highly efficient adjuvant upon influenza vaccination in different aged groups, i.e., young neonates, infants, adults, and old aged mouse models. The adjuvant effects of BCG-CWS are related to the improvement of immunogenicity and protective efficacy of influenza vaccines and amelioration of pathological inflammation, in all aged groups of mice. In addition, the present results provide strong evidence that BCG-CWS is a crucial contributor to the adjuvant activity for influenza vaccination through protective adaptive immune responses and APC function in diverse age populations.

BCG vaccination in infants provides better survival advantages against various pathogens, not specific to *M. tuberculosis* [3,5]. In addition, BCG-CWS can be used as vaccine adjuvants, although it has been less studied as compared to its use in cancer immune therapies [17,40,41]. Oil-in-water emulsion itself has adjuvant effects in several infection models. Subcutaneous vaccination of mice with recombinant hepatitis B surface protein (25 µg) in and oil-in-water emulsion of high dose BCG-CWS (100 µg/mouse) is sufficient to increase the IgG1 isotype-manifested Th2 humoral and cellular responses [18]. Conjugation of BCG-CWS with ovalbumin as a model protein antigen enhances the immunogenicity of conjugated antigens toward a Th1-predominant response with production of IgG antibodies and induction of cellular responses [21]. However, chemical conjugating an antigen to BCG-CWS might shield the effects of functional and protective epitopes in vaccine antigens. Thus, we investigated the adjuvant effects of free BCG-CWS mixed with split vaccine, a most common platform of influenza vaccination. BCG-CWS was significantly more effective in the induction of IgG1 and IgG2a antibodies and cross-protection after influenza poorly immunogenic protein (5xM2e) vaccine than aluminum adjuvants biasing Th2 type IgG1 isotype antibody responses (data not shown). Surprisingly, BCG-CWS adjuvant effects were evident in the enhancement of IgG1 and IgG2a antibodies and HAI titers by single prime dose influenza vaccine in adult mice. In addition, BCG-CWS showed a marked adjuvant effect on influenza vaccination such as lung viral clearance and prevention of weight loss, and attenuation of inflammation in vaccinated mice. In addition, BCG-CWS adjuvant appears to enhance IgG1 and IgG2a responses.

Antigen exposure in neonates and infants often results in undetectable, low, or delayed IgG antibody responses [30,42,43]. Nonetheless, adult-like immune responses can be induced in early life under a specific condition of immune stimulation [44]. This study provides convincing evidence that BCG-CWS exhibits significant adjuvant effects on the enhancement of the immunogenicity and protective efficacy in young age (6 D and 2 W) mice when boosting with vaccines. In contrast to adult mice, an optimal boost dose is likely required to induce comparable protective immunity in young infant age mice. Neonates tend to induce T helper type 2 (Th2) responses, which is presumably related to epigenetic hypomethylation at the loci of Th2 cytokines [45,46]. Consistently, Th1 type IgG2a antibody induction was not observed in 7 D-age mice, but significant levels of IgG2a were induced in 2 W-age mice, after BCG-CWS adjuvanted vaccination. In addition, BCG-CWS adjuvanted vaccination in infant age mice prevented inflammatory infiltrates such as eosinophils and monocytes in the lungs and BALF after lethal infection. Moreover, BCG-CWS adjuvanted vaccination markedly decreased the proinflammatory cytokine generation in the infection sites in all aged groups of mice. Strategies employed to develop efficient vaccines and adjuvant depend on appropriate induction of innate immune and antigen-specific immune responses to upregulate protective defense and immune memory [47,48]. In addition, age-related intrinsic inflammation with decreased immune function should be considered in the design of influenza vaccines and adjuvants [23]. Consistent, young age mice with BCG-CWS adjuvanted vaccination alleviated histopathology due to lethal influenza infection (data not shown). Importantly, the results presented here indicate that BCG-CWS adjuvanted influenza vaccination would prevent pathological inflammation of cytokines and infiltrates in the infection sites, and a successful activator of immunogenicity and protective immune responses to influenza vaccination in all aged groups of mice.

The extremes of age are related to the increased infectious rates and severity of infections [30]. Aging often results in severe defects in the generation of new lymphocytes and protective immune responses to vaccination [25,26,27,30]. It is highly challenging to enhance the vaccine efficacy in the naïve elderly population due to declining ability to generate adaptive immune responses [28,29]. It should be noted that split vaccine only, in 6 D- and 2 W-infant aged mice, generates adult-like memory B cells which rapidly differentiate into virus-specific IgG antibodies upon infection (Figure 6D–F). This is in line with a study that early life antigen exposures rarely induce marked antibody responses but can prime the B cells for enhanced antibody responses after subsequent challenge [49]. Consistent with previous studies on aging immune responses [23,24], split vaccination of 20 M-aged mice failed to induce virus-specific IgG antibodies at a meaningful level even after boost, in contrast to those in 6 D and 2 W mice. This defect is further evident as limited IgG responses in split vaccine only in 20 M mice upon virus challenge, whereas the 6 D, 2 W, and 6 W age mice with split vaccination induced high levels of IgG, after challenge infection. However, inclusion of BCG-CWS in split vaccination of 20 M-aged mice significantly enhanced the levels of IgG1 and IgG2a isotypes, HAI titers, protective efficacy, and viral clearance, suggesting successful amplification of protective humoral and cellular immunity. A large body of evidence shows aging-related defects in CD4^+^ T cell function to contribute to low vaccine efficacy in the elderly [23,50]. In this aspect, it is remarkable that BCG-CWS adjuvanted vaccination significantly promoted the induction of IFN-γ^+^CD4^+^ T cells even in 20 M-aged mice.

Our data indicate that BCG-CWS adjuvant effects are related to innate immune activation by stimulation of APCs such as macrophage and dendritic cells. We found that BCG-CWS stimulates BMDMs and BMDCs to secrete proinflammatory cytokines (TNF-α and IL-6), Th1 type IL-12, and regulatory IL-10 cytokines, and that these effects were, at least partly, mediated through TLR2 and TLR4. Our data correlated with previous findings that BCG-CWS induces immune responses through TLR2 and TLR4 [20]. In addition, BCG-CWS-induced cytokine generation is dependent on the myeloid differentiation factor (MyD88) in BMDCs (data not shown). These data are partly consistent with a previous study showing that BCG-CWS induces anticancer effects through TLR2 and TLR4 [17], as well as MyD88 [51]. “Trained immunity” of non-specific protection was reported with prior live BCG vaccination [4,5,6,7], which might be associated with autophagy activation [52]. In contrast, the BCG-CWS adjuvant injection without vaccine antigen did not show any protection in mice under the lethal challenge condition used in this study, suggesting that enhanced protection was resulted from the adjuvant effects on adaptive immunity rather than non-specific protection.

In summary, our data highlight the effects of BCG-CWS as adjuvants of influenza vaccines for different aged groups of mice through enhancement of the immunogenicity and protective efficacy of vaccination. BCG-CWS exhibits multifunctional effects including protective humoral and cellular immune responses, control of excessive pathological inflammation, and Th1 cell proliferation upon influenza challenge. Mechanistically, BCG-CWS adjuvants effects upon APC function is mediated through TLR2 and 4 signaling pathways. These data strongly suggest that vaccine adjuvant BCG-CWS can be useful as novel adjuvants to overcome the hurdles in defective vaccine responses, often observed in the extreme aged population. Recent studies implicate that prior BCG vaccination is associated with positive correlation with protection against COVID-19, in which the mortality rate is highly associated with old age [6,7,53]. We believe that the current findings will facilitate the BCG-CWS-based development of future vaccine/adjuvant formulation, not only to influenza, but also to various infections such as COVID-19.

## Data Availability

All data that support the findings of this study are available from the corresponding author upon reasonable request.

## Supplementary Materials

The following are available online at https://www.mdpi.com/article/10.3390/biomedicines9050516/s1. Supplementary Figure S1. The frequency of inflammatory and antigen presenting cells in the lungs after challenge infection. Table S1. Cytokine levels after in vitro stimulation of BMDMs and BMDCs from wild type BALB/C and C57BL/6 mice.

## Abbreviations

BCG	Bacillus Calmette-Guerin
CWS	cell wall skeleton
TLR	Toll-like receptors
DCs	dendritic cells
TNF-α	tumor necrosis factor-αlpha
IL	interleukin
BALF	bronchoalveolar lavage fluid
BMDMs	bone marrow derived macrophages
BMDCs	bone marrow derived dendritic cells
APCs	antigen presenting cells
MyD	myeloid differentiation factor
Th1	T helper type 1
Th2	T helper type 2
