# Supplementary material for: BCG Cell Wall Skeleton As a Vaccine Adjuvant Protects Both Infant and Old-Aged Mice from Influenza Virus Infection
Source: Biomedicines. 2021 May 5;9(5):516. doi: 10.3390/biomedicines9050516 (PMC8148143; doi:10.3390/biomedicines9050516)
Supplement: Supplementary file 1 [file biomedicines-09-00516-s001.zip › biomedicines-1194010-supplementary.pdf]

biomedicines

BCG Cell Wall Skeleton As a Vaccine Adjuvant Protects Both Infant and Old-Aged Mice from Influenza Virus Infection

Ki-Hye Kim [1], Young-Tae Lee [1], Yoonsuh Park [1], Eun-Ju Ko [1,2], Yu-Jin Jung [1], Yu-Jin Kim [1,3], Eun-Kyeong Jo [4,5] and Sang-Moo Kang [1,*]

Supplementary Information

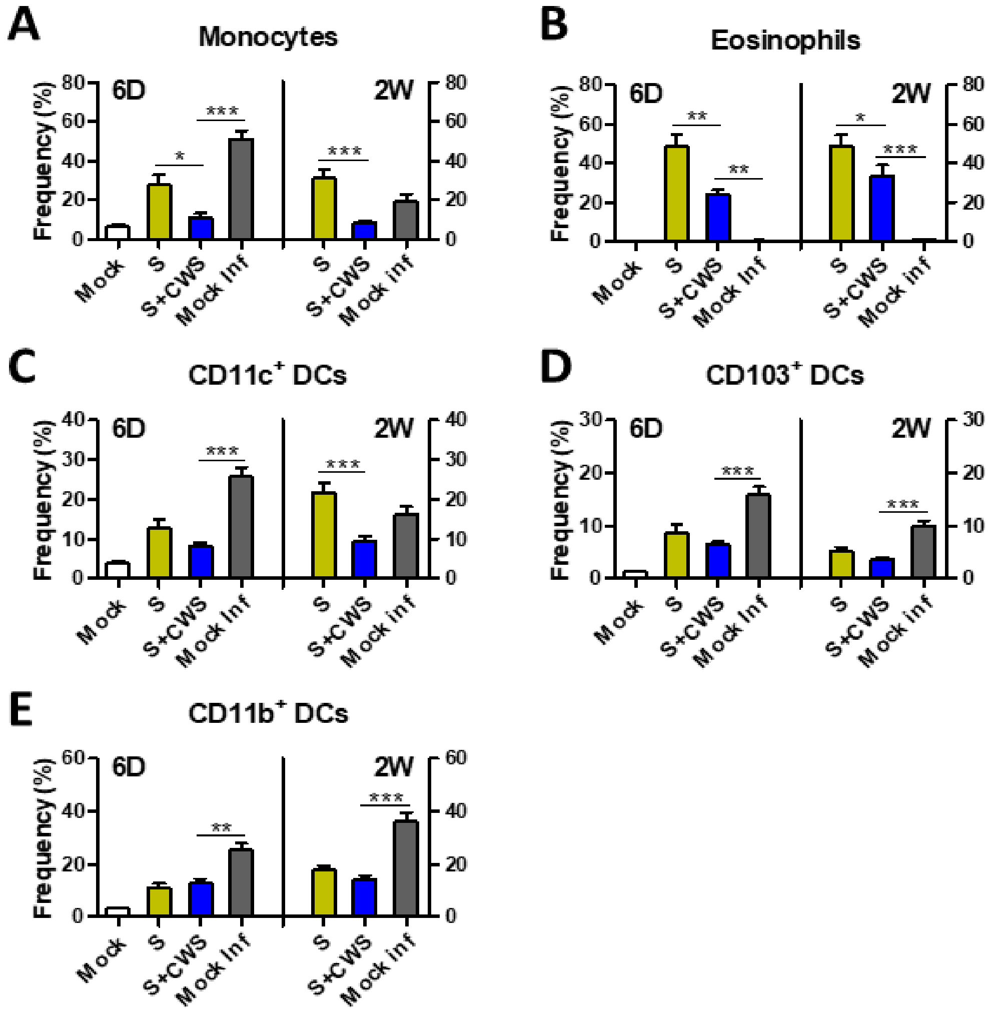

Figure S1. The frequency of inflammatory and antigen presenting cells in the lungs after challenge infection. Cellular phenotypes were determined in the lung samples collected at 7 dpi with A/Cal virus by flow cytometry. (A-B) Both monocytes and eosinophils are presented in percentages out of the total gated $CD11b^+$ cells in lung cells. (A) Monocytes ($CD11b^+Ly6c^{hi}F4/80^+$), (B) Eosinophils ($CD11b^+CD11c^-SiglecF^+$). (C) $CD11c^+$ dendritic cells (DCs, $CD45^+$ $F4/80^-$ $CD11c^+MHCII^{hi}$) presented in percentages out of total gated $CD45^+F4/80^-$ cells in lung cells. (D and E) $CD103^+$ DCs ($CD45^+F4/80^-CD11c^+MHCII^{hi}CD11b^-CD103^+$) and $CD11b^+$ DCs ($CD45^+F4/80^-CD11c^+MHCII^{hi}CD11b^+CD103^-$) are presented in percentages out of the total gated $CD45^+F4/80^-CD11c^+MHCII^{hi}$ cells in the Lung of 6D and 2W age mice. Statistical significance was calculated by using one-way ANOVA and a Dunnett's multiple-comparison test. Error bars indicate the mean ± SEM. *; $p<0.05$, **; $p<0.01$, ***; $p<0.001$.

Table S1. Cytokine levels after *in vitro* stimulation of BMDMs and BMDCs from wild type BALB/C and C57BL/6 mice. Cytokines in culture supernatants were presented in concentrations (pg/ml) after incubation of BMDCs and BMDMswith a stimulator, medium control or BCG-CWS (10 μg/ml) for 24 hours.

Table 1. Cytokine levels after *in vitro* stimulation of BMDCs and BMDMs

Mice	BALB/c								C57BL6		
Cell	BMDCs				BMDMs				BMDMs		
Cytokines (pg/mL)	TNF-α	IL-6	IL-12	IL-10	TNF-α	IL-6	IL-12	IL-10	TNF-α	IL-6	IL-12
Control	-	-	-	-	-	-	-	-	-	-	-
CWS (10μg/ml)	4,597.4 ± 563.1	4,163.8 ± 465.4	1,301.3 ± 197.2	1,190.4 ± 184.2	5,457.9 ± 66.6	2,298.8 ± 204.8	472.8 ± 89.8	241 ± 10.4	6471.2 ± 800.7	3586.7 ± 104.8	874.7 ± 35.2
